# Relationship of the gene pool of the Khants with the peoples of Western Siberia, Cis-Urals and the Altai-Sayan Region according to the data on the polymorphism of autosomic locus and the Y-chromosome

**DOI:** 10.18699/VJGB-23-07

**Published:** 2023-03

**Authors:** V.N. Kharkov, N.A. Kolesnikov, L.V. Valikhova, A.A. Zarubin, M.G. Svarovskaya, A.V. Marusin, I.Yu. Khitrinskaya, V.A. Stepanov

**Affiliations:** Research Institute of Medical Genetics, Tomsk National Research Medical Center of the Russian Academy of Sciences, Tomsk, Russia; Research Institute of Medical Genetics, Tomsk National Research Medical Center of the Russian Academy of Sciences, Tomsk, Russia; Research Institute of Medical Genetics, Tomsk National Research Medical Center of the Russian Academy of Sciences, Tomsk, Russia; Research Institute of Medical Genetics, Tomsk National Research Medical Center of the Russian Academy of Sciences, Tomsk, Russia; Research Institute of Medical Genetics, Tomsk National Research Medical Center of the Russian Academy of Sciences, Tomsk, Russia; Research Institute of Medical Genetics, Tomsk National Research Medical Center of the Russian Academy of Sciences, Tomsk, Russia; Research Institute of Medical Genetics, Tomsk National Research Medical Center of the Russian Academy of Sciences, Tomsk, Russia; Research Institute of Medical Genetics, Tomsk National Research Medical Center of the Russian Academy of Sciences, Tomsk, Russia

**Keywords:** gene pool, human population, genetic diversity, genetic components, Y-chromosome, Khanty, генофонд, популяции человека, генетическое разнообразие, генетические компоненты, Y-хромосома, ханты

## Abstract

Khanty are indigenous Siberian people living on the territory of Western Siberia, mainly on the territory of the Khanty-Mansiysk and Yamalo-Nenets Autonomous Okrugs. The present study is aimed at a comprehensive analysis of the structure of the Khanty gene pool and their comparison with other populations of the indigenous population of Southern and Western Siberia. To address the issues of genetic proximity of the Khanty with other indigenous peoples, we performed genotyping of a wide genomic set of autosomal markers using high-density biochips, as well as an expanded set of SNP and STR markers of the Y-chromosome in various ethnic groups: Khakas, Tuvans, Southern Altaians, Siberian Tatars, Chulyms (Turkic language family) and Kets (Yeniseian language family). The structure of the gene pool of the Khanty and other West Siberian and South Siberian populations was studied using a genome-wide panel of autosomal single nucleotide polymorphic markers and Y-chromosome markers. The results of the analysis of autosomal SNPs frequencies by various methods, the similarities in the composition of the Y-chromosome haplogroups and YSTR haplotypes indicate that the Khanty gene pool is quite specific. When analyzing autosomal SNPs, the Ugrian genetic component completely dominates in both samples (up to 99–100 %). The samples of the Khanty showed the maximum match in IBD blocks with each other, with a sample of the Kets, Chulyms, Tuvans, Tomsk Tatars, Khakas, Kachins, and Southern Altaians. The degree of coincidence of IBD blocks between the Khanty, Kets, and Tomsk Tatars is consistent with the results of the distribution of allele frequencies and common genetic components in these populations. According to the composition of the Y-chromosome haplogroups, the two samples of the Khanty differ significantly from each other. A detailed phylogenetic analysis of various Y-chromosome haplogroups made it possible to describe and clarify the differences in the phylogeny and structure of individual ethnospecific sublines, to determine their relationship, traces of population expansion in the Khanty gene pool. Variants of different haplogroups of the Y-chromosome in the Khanty, Khakas and Tuvans go back to their common ancestral lines. The results of a comparative analysis of male samples indicate a close genetic relationship between the Khanty and Nenets, Komi, Udmurts and Kets. The specificity of haplotypes, the discovery of various terminal SNPs confirms that the Khanty did not come into contact with other ethnic groups for a long time, except for the Nenets, which included many Khanty clans.

## Abstract (ru)

Ханты – коренной сибирский народ, проживающий на территории Западной Сибири, в основном на территории Ханты-Мансийского и Ямало-Ненецкого автономных округов. Настоящее исследование направлено на комплексный анализ структуры генофонда хантов и их сравнение с другими популяциями коренного населения Южной и Западной Сибири. Для решения вопросов генетической близости хантов с другими коренными народами выполнено генотипирование широкого геномного набора аутосомных маркеров с помощью высокоплотных биочипов, а также расширенного набора SNP- и STR-маркеров Y-хромосомы у различных эт- нических групп: хакасов, тувинцев, южных алтайцев, сибирских татар, чулымцев (тюркская языковая семья) и кетов (енисейская языковая семья). Результаты анализа частот аутосомных SNP различными методами, сход- ства по составу гаплогрупп Y-хромосомы и YSTR-гаплотипов свидетельствуют, что генофонд хантов достаточно специфичен. При анализе аутосомных SNP в обеих выборках полностью доминирует угорский генетический компонент (до 99–100 %). Выборки хантов показали максимальное совпадение по IBD-блокам между собой, с выборкой кетов, чулымцев, тувинцев, томских татар, хакасов-качинцев и южных алтайцев. Степень совпа- дения IBD-блоков между хантами, кетами и томскими татарами согласуется с результатами распределения в этих популяциях частот аллелей и общих генетических компонентов. По составу гаплогрупп Y-хромосомы две выборки хантов значительно различаются между собой. Детальный филогенетический анализ различных га- плогрупп Y-хромосомы позволил описать и уточнить различия в филогении и структуре отдельных этноспе- цифичных сублиний, определить их родство, следы экспансии численности в генофонде хантов. Варианты разных гаплогрупп Y-хромосомы у хантов, хакасов и тувинцев восходят к общим для них предковым линиям. Результаты сравнительного анализа образцов мужчин также свидетельствуют о близком генетическом родстве между хантами и ненцами, коми, удмуртами и кетами. Специфичность гаплотипов, обнаружение различных терминальных SNP подтверждают, что ханты достаточно долго не имели контактов с другими этносами, кроме ненцев, в состав которых вошло много хантыйских родов.

## Introduction

The study of the structure of the gene pools of populations of various Siberian regions is one of the priority areas of modern human genetics and helps to reveal in detail some of the issues related to their ethnogenesis

The Khanty are an indigenous people living on the territory of Western Siberia, mainly on the territory of the Khanty-Mansiysk and Yamalo-Nenets Autonomous Okrugs, as well as the Tyumen Region. Small groups of Khanty live in the north of the Tomsk Region and in the Komi Republic. According to the All-Russian census of 2010, the number of Khanty was 30,943 people, of which 61.6 % lived in the Khanty-Mansi Autonomous Okrug and 30.7 %, in the Yamalo-Nenets Autonomous Okrug. The Khanty have three large ethnographic groups that coincide with the groups of their language dialects – northern, southern and eastern, and the southern (Irtysh) Khanty were Turkified and became part of the Siberian Tatars, having mixed with them, and were also assimilated by Russian settlers (Peoples of West Siberia…, 2005).

Khanty populations are of considerable interest for population genetic studies, both due to the relatively poor knowledge with the involvement of modern genomic technologies, and due to the specificity of the gene pools of their individual groups that developed under conditions of long-term genetic isolation.

The settlement of the Khanty in antiquity was very wide – from the lower reaches of the Ob in the north to the Baraba steppes in the south and from the Yenisey in the East to the Trans-Urals, including the rivers Northern Sosva and Lyapin, as well as part of the rivers Pelym and Konda in the west. Since the 19th century, the Mansi began to move beyond the Urals from the Kama and Ural regions, being pressed by the Komi-Zyryans and Russians. From an earlier time, part of the southern Mansi also left to the north in connection with the creation in the XIV–XV centuries of the Tyumen and Siberian khanates – the states of the Siberian Tatars, and later (XVI– XVII centuries) with the development of Siberia by the Russians. In the XVII–XVIII centuries, the Mansi already lived on Pelym and Konda. Part of the Khanty also moved from the western regions to the east and north (to the Ob from its left tributaries), which is recorded by the statistical data of the archives. Their place was taken by the Mansi. So, by the end of the XIX century, there was no Ostyak population left on the rivers Northern Sosva and Lyapin: they either moved to the Ob or merged with the newcomers (The Peoples of Russia, 1994).

In the north, the Khanty came into contact with the Nenets, some of them were assimilated by them, which is confirmed by ethnographic data, as well as our study of the tribal structure of the Gydan Nenets according to Y-chromosome markers (Kharkov et al., 2021). The migration of the Khanty to the north and east continued into the 20th century. By the 20th century, the southern Khanty were almost completely assimilated by the Tatars and Russians

Historically, the Khanty population was not homogeneous either in language or culture. Some scientists divide the Khanty language into two large groups – western and eastern, while others still subdivide the western dialects into southern and northern. In anthropological terms, the Khanty are the most characteristic representatives of the Ural type, which also includes the Mansi, Selkups, Nenets, Baraba Tatars, Shors, Northern Altaians and Khakas. The closest relatives of the Khanty in origin, language and culture are the Mansi (Brook, 1986).

The purpose of this study is a comprehensive analysis of the structure of the Khanty gene pool and the reconstruction of their origin in comparison with other populations of the indigenous population of Southern and Western Siberia. To address the issues of genetic proximity of the Khanty with other indigenous peoples, genotyping of a wide genomic set of autosomal markers using high-density biochips, as well as an expanded set of SNP and STR-markers of the Y-chromosome was performed in various ethnic groups: Khakas, Tuvans, Southern Altaians, Siberian Tatars, Chulyms (Turkic language family) and Kets (Yeniseian language family).

## Materials and methods

The material of the study was DNA samples of men and women from two populations of the Khanty in the village of Russkinskaya, Surgut district and the village of Kazym, Beloyarsky district of the Khanty-Mansi Autonomous Okrug. The sampling of primary biological material (venous blood) from donors was carried out in compliance with the procedure of written informed consent for the study. For each donor, a questionnaire was compiled with a brief pedigree, indicating ethnicity and places of birth of ancestors. An individual was assigned to a given ethnic group based on their own ethnic identity, their parents and place of birth.

For the analysis of Y-chromosome haplogroups and haplotypes of the Khanty, 120 DNA samples of men from the village of Russkinskaya (N = 64) and the village of Kazym (N = 54) of the Khanty-Mansi Autonomous Okrug were used. For genotyping on high-density microchips, unrelated samples from the village of Kazym (N = 30) and the village of Russkinskaya (N = 26) were selected. Other populations of the indigenous population of Siberia are represented by: Chulyms (N = 22), Khakas (Sagays of the Tashtyp district, N = 29 and Kachins of the Shirinsky district, N = 26), Southern Altaians (village of Beshpeltir of the Chemal district, N = 24 and Kulada village, Ongudaysky district, N = 25), Kets (Kellogg village, Turukhansky district, Krasnoyarsk Territory, N = 15), Tomsk Tatars (Chernaya Rechka village, Eushta village and Takhtamyshevo village, Tomsky district, N = 20), Tuvinians (Teeli village of Bai-Taiginsky kozhuun, N = 28).

Genome-wide genotyping data were obtained using Infinium Multi-Ethnic Global-8 (Illumina) microarrays for SNP genotyping, including over 1.7 million markers. The material was deposited in the bioresource collection “Biobank of the Population of Northern Eurasia”.

Autosomal SNP (single nucleotide polymorphism) genotype array clustering and quality control were performed using a protocol developed by (Guo et all., 2014) using GenomeStudio (Ilumina. GenomeStudio) (genotyping module v2.0.3), a software package that Illumina developed for various genomic analyses. For filtering, normalizing and calculating standard genomic statistics and indicators, the standard set of programs, including vcftools, bcftools, and plink, proved to be optimal.

To analyze linkage blocks identical in origin, the Refined IBD algorithm (Browning B.L., Browning S.R., 2013) was used, which shows more accurate results compared to the algorithms built into plink. The genotypes were preliminarily phased using the Beagle 5.1 software (Browning S.R., Browning B.L., 2007). To compare the populations, the sums of the average lengths of blocks identical in origin (IBD segments – identical by descent) were obtained between pairs of individuals.

The tSNE method was used to analyze genetic relationships between populations. The NGSadmix method (Scotte et al., 2013) and the ADMIXTURE program (Alexander et al., 2009; Alexander, Lange, 2011) were used to analyze the component composition and the amount of impurities in individuals and populations

To study the composition and structure of Y-chromosome haplogroups, two systems of genetic markers were included in the study: diallelic locuses represented by SNPs and polyallelic highly variable microsatellites (YSTRs). With the help of 138 SNP markers, the belonging of the samples to different haplogroups was determined. The classification of haplogroups is given in accordance with the data of the International Society for Genetic Genealogy (website www.isogg.org).

Analysis of STR haplotypes within haplogroups was performed using 44 STR markers of the non-recombining part of the Y chromosome (DYS19, 385a, 385b, 388, 389I, 389II, 390, 391, 392, 393, 426, 434, 435, 436, 437, 438, 439, 442, 444, 445, 448, 449, 456, 458, 460, 461, 481, 504, 505, 518, 525, 531, 533, 537, 552, 570, 576, 635, 643, YCAIIa, YCAIIb, GATA H4.1, Y-GATA-A10, GGAAT1B07). STR markers were genotyped using capillary electrophoresis on an ABI Prism 3730 genetic analyzer. Genotyping of SNP markers was performed using PCR and subsequent analysis of DNA fragments using RFLP analysis.

Experimental studies were carried out on the basis of the Center for the Collective Use of Research Equipment “Medical Genomics” (Research Institute of Medical Genetics of the Tomsk National Research Medical Center). The construction of median networks of Y-chromosome haplotypes was carried out using the Network v.10.2.0.0 (Fluxus Technology Ltd; www.fluxus-engineering.com) using the Bandelt median network method (Bandelt et al., 1999). The generation age of the observed diversity of haplotypes in haplogroups was estimated using the ASD method (Zhivotovsky et al., 2004) based on the mean square differences in the number of repeats between all markers

## Results and discussion

The large array of data on autosomal SNPs obtained as a result of genotyping of high-density microarrays in samples of the Khanty and other indigenous Siberian peoples makes it possible to characterize the gene pool of the studied samples in the most detailed way using various methods. Genotyping of an extended set of specific Y-chromosome SNPs from various haplogroups makes it possible to describe the molecularphylogenetic and phylogeographic structure of individual Y-chromosome haplogroups much more accurately.

After processing the data on the results of a microarray study to filter the progenotyped samples and perform further calculations, a search was carried out among the mestizo Khanty using the NGSadmix program. The algorithm of this program makes it possible to determine the ratio of ancestral components from NGS data with a relatively shallow coverage depth. The calculation principle is similar to other programs such as FRAPPE and ADMIXTURE, but NGSadmix, unlike them, works effectively when there is statistical uncertainty in individual genotypes. The NGSadmix method, when run on the data array we formed, showed that almost all Khanty samples do not have crossbreeding, which is fully consistent with the data from the DNA donor questionnaire. Crossbreeding with Russians (up to 30 %) was found only for one man from the village of Russkinskaya. His belonging to the European Y-chromosomal lineage R1b1a1b-L407 confirms the miscegenation on the paternal side. This sample was excluded from further calculations.

The obtained data on the frequencies of SNPs in the studied samples were used to elucidate the genetic relationships between the population samples included in the work. For dimensionality reduction, spatial analysis, and identification of genetic components, we settled on two algorithms: tSNE and ADMIXTURE. The tSNE method makes it possible to more clearly divide the data array into separate ethnospecific groups of samples compared to the PCA method.

Genetic relationships of the Khanty with other populations of Western and Southern Siberia

**Fig. 1.**
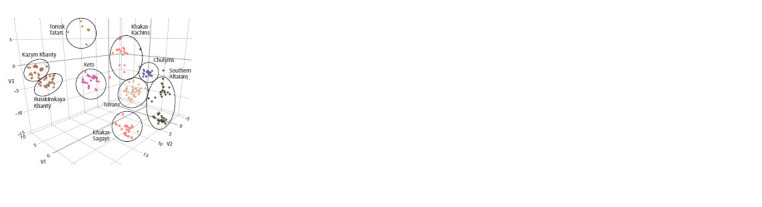
Differentiation of the genomes of the population of Southern and Western Siberia by three components of tSNE.

When analyzing the data array on the frequencies of autosomal SNPs using the tSNE method at the level of individual samples (Fig. 1). It is shown that the two samples of the Khanty are very close, while the samples of the Kazym and Russkinskaya Khanty do not intersect on the graph and are separated from each other

The Khanty are characterized by specific features of the gene pool and do not cluster with other populations. Compared with subethnic groups of the Khakas and Southern Altaians from different settlements, more geographically distant samples of the Khanty demonstrate a much greater genetic closeness. The samples of the Kets and Tomsk Tatars are closest to the Khanty. The genetic distances between the Khanty and the populations of Southern Siberia are much greater. Samples that are ethnically and geographically close to each other are located quite close in the Fig. 1, but each sample is included in a separate ethnospecific cluster. The exception is only a few single samples of the Khakas.

Component composition of the gene pool of populations. Modern methods used in genomic studies and new bioinformatic approaches make it possible to reliably identify ancestral genetic components of different origins in the gene pool of various populations and individuals. To identify individual genetic components in the gene pool of the studied populations, the ADMIXTURE program was used, which makes it possible to identify the mixed composition of a set of individuals based on genotype data and, thereby, to make assumptions about the origin of the population.

Modeling using ADMIXTURE has recently become one of the main methods of analysis in the study of the gene pools of modern and ancient human populations, allowing you to analyze the same data at different hierarchical levels. When the number of ancestral components is set to more than two, in most of the studied populations, a genetic component specific to the Khanty is revealed, which is most clearly manifested in the analyzed array of population samples at K = 8, which can be interpreted as the “Ugric” genetic layer in the gene pool of modern populations. The Khanty are characterized by the dominance of this component, which is their genetic basis (up to 99–100 % at the level of most individuals). A significant proportion of this component is also found in the Kets (up to 45–50 % in some individuals) and Tomsk Tatars (up to 5–9 %). Previously, it was shown that this component also occupies a significant share in the gene pool of the populations of the Volga-Ural region – the Bashkirs (up to 25 %), Maris (up to 20 %), Komi, Udmurts and Chuvashs (up to 15 %). It is present with less frequency in almost all South Siberian samples, among the Tuvans, Chulyms, Altaians, and Khakas of Sagays (from 5 to 10 %) (Kharkov et al., 2020).

The dominance of the Ugric component in all Khanty samples, starting from K = 3, and the almost complete absence of other genetic components in their genomes at the individual and population level, indicates that their ancestral populations were in genetic isolation for a very long time. This suggests that the ancient Ugric population of the modern territory of the Khanty settlement did not mix with other ethnic groups and confirms the absence of other groups of migrants from the territory of Southern Siberia and the steppe zone

The result obtained shows that the overall picture of the distribution of the components is in good agreement with the geographical location of the studied populations, binding to a specific region, anthropological and linguistic differences. This information makes it possible to more accurately judge the similarities and differences between the compared populations, the composition of ancestral components, as well as the process of formation of their gene pool.

Identical in origin clutch blocks. As a result of bioinformatics processing of genotyping data from high-density biochips of various Siberian populations, an analysis was made of the coincidence of DNA fragments common in origin between populations and individuals. A segment with identical nucleotide sequences is IBD in two or more individuals if they inherit it from a common ancestor without recombination, that is, in these individuals the segment has a common origin. The expected length of an IBD segment depends on the number of generations since the last common ancestor. One of the applications of the analysis of genome regions of common origin is the quantitative assessment of the degree of relationship between individuals, which can also supplement information on the genetic relationships of populations (Gusev et al., 2012).

The samples of the Khanty showed the maximum match in IBD blocks with each other (6 %), then with a sample of the Kets (1.45 %), Chulyms (0.71 %), Tuvans (0.35 %), Tomsk Tatars (0.33 %), Khakas Kachins (0.32 %), and Southern Altaians (0.28 %). At the same time, among the Khanty, a greater coincidence of IBD blocks is observed in Russkinskaya (23.5 %), compared with Kazym (18.1 %).

The degree of overlap of IBD blocks between the Khanty, Kets, and Tomsk Tatars is consistent with the results of tSNE and ADMIXTURE in terms of the distribution of allele frequencies and common genetic components in these populations. At the same time, in Khanty population from Russkinskaya, who have the largest sum of average lengths of IBD segments between pairs of individuals, the greatest contribution is made by IBD longer than 10 cm (42–46 %), which indicates a strong recent inbreeding within the population. To confirm this, the FROH inbreeding coefficient was calculated for all individuals for the three classes of homozygosity blocks (ROH). For the West Siberian populations, the Chu- lym population (0.0292), the Kazym (0.0280) and Russkinskaya Khanty (0.0266) and Kets (0.0259) populations, which are close in value, have the maximum values. Among the South Siberian populations, including the Altaians, Tomsk Tatars, Tuvans and Khakas, the maximum value was also found for the sample of Khakas-Sagays from the foothill Tashtyp region (0.0318), twice as high as the Khakas-Kachins of the plain Shirinsky region. The minimum value is typical for the Tomsk Tatars (0.0071).

There is a high correlation for FROH > 1.5 with the sum of mean IBD segment lengths (IBD > 1.5 cM) between pairs of individuals within Siberian populations (r = 0.9246, p < 5.612e-09). To calculate the Spearman correlation coefficient, cor.test was used in the R program. The ratio of the sum of the average lengths of IBD segments (IBD > 1.5 cM) between pairs of individuals to the coefficient of genomic inbreeding (FROH > 1.5) in the Russkinsskaya Khanty is higher than in Kazym Khanty. These indicators of genomic inbreeding and distribution of IBD lengths within Khanty populations are in good agreement with their territorial isolation and confirm the absence of recent gene flows between populations for hundreds of years

Haplogroups of the Y-chromosome. As a result of the analysis of the frequency of occurrence of the used SNP markers in the studied samples of the Khanty, eight haplogroups of the Y-chromosome were identified. According to the composition and frequencies of haplogroups, the samples of Russian and Kazym Khanty men are very different from each other. Only two haplogroups are present in both samples (see the Table).

**Table 1.**
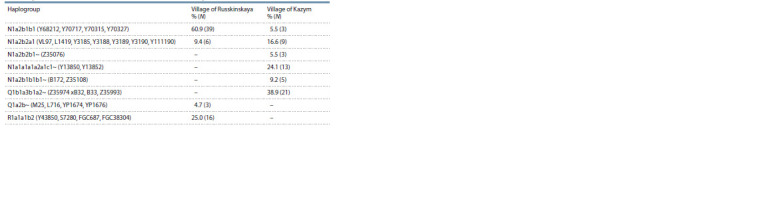
Frequency of occurrence of Y-chromosome haplogroups in the Khanty

Thirty-nine samples belong to the N1a2b1b1 subline in the Russkinskaya Khanty, and only three in the Kazym ones. Terminal for this line, the Khanty have SNPs Y68212, Y70717, Y70315, Y70327. This Khanty subline is close to the N1a2b1b1 variants in the Chulyms (VL65, Z35095, Z35099, Z35102) and Khakas-Kachins (Z35093, Z35097, Z35103) (Valikhova et al., 2022).

The haplogroup N1a2b1b1 among the Khanty is ethnospecific and does not coincide in terminal SNPs and haplotypes with the dominant among the Nenets N1a2b1b1a~ (B171, B170, Z35091, Z35092) (Kharkov et al., 2021).

**Fig. 2.**
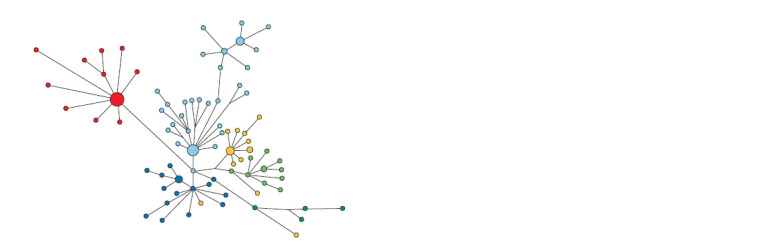
The median network of YSTR haplotypes of the N1a2b1b1 haplogroup in the Khanty, Chulyms and Khakas-Kachins. The Khanty are marked in light blue, the Chulyms are in red, the Khakas of the Sokhkhy seok are in blue, the Khakas of the Yzyr seok are in green, and the yellow are Khakases seok Hhaskha, dark green – seok Purut.

A feature of the ethnic composition of the majority of the South Siberian peoples is the presence of clans (seoks), where kinship is counted along the male line. Such a generic structure is typical for the Shors, Khakas, northern and southern Altaians, and Teleuts. All other samples of men from various West and South Siberian populations (the Enets, Khakas- Sagays, Shors, Chelkans and Tuvans, as well as the Khakas seoks formerly part of the Beltirs and Biryusins, assimilated in the late 19th and early 20th centuries) belong to others sublines of haplogroup N1a2b. The median network of haplotypes (Fig. 2) demonstrates a stellate phylogeny in the Khanty with a recent founder effect and a predominance of the ancestral haplotype in frequency.

The specific cluster of Khanty haplotypes is equidistant from all seoks of the Khakas-Kachins. The age of this cluster among the Khanty was 858 years (SD = 338 years), which is approximately one and a half to two times higher than the age of the clusters of the Kachin seoks Khaskha – 487 years (SD = 153 years), Yzyr – 501 years (SD = 203 years), Sokhkhy – 585 years (SD = 215 years) (Kharkov et al., 2020) and Chulym Turks 667 years (SD = 194 years). Thus, the Khanty in this haplogroup have a direct genetic connection with the Kachins, Chulyms and Nenets, whose ancestral lines diverged quite a long time ago and reflect their connection with the peoples of the Samoyedic language group.

The second haplogroup N1a2b2a1 (VL97, L1419, Y3185, Y3188, Y3189, Y3190, Y111190) is common for two Khanty samples (previously designated as the European N1b-E lineage). This subline was found among the Bashkirs, Kazan Tatars, Komi, Mari, Karelians, Vepsians, Finns and Russians (https://www.yfull.com/). Phylogenetically closest to the Khanty along this line are the Komi samples. Ethnospecific branches of the Khanty and Komi unite SNPs Y65017 and Y89655, not found in other populations. The Khanty and Komi have the least ancient common ancestor for this haplogroup, compared to other European populations.

According to the YFull website, this branch split from the ancestral line about 2800 years ago. Theoretically, there are two options for the appearance of this haplogroup among the modern Khanty and Komi: 1) inheritance from a common ancient ancestral group of Ugric tribes; 2) the recent mixing of Khanty with ethnic Komi migrants to Siberia. However, the results of the analysis of genomic data using NGSadmix, ADMIXTURE, IBD blocks and differences in terminal SNPs of Y-chromosomes do not confirm the second variant. The YSTR haplotypes of this line in the Khanty and Komi also differ by several mutations. Previously, V.N. Pimenoff et al. suggested in their work that when the Ob-Ugric Khanty and Mansi went to the western slopes of the Ural Mountains and to the north-west of Siberia, a unique association N1b-A and N1b-E was formed (Pimenoff et al., 2008). This combination of N1b sublines in the Khanty and Mansi suggests a recent confluence of the western and eastern lineages in North Western Siberia. Our new data do not contradict this version

All other haplogroups are represented only in individual samples of the Khanty. The haplogroup N1a2b2b1~ (Z35076) includes three samples of the Kazym Khanty. The lineage N1a2b2b1~ (B528, Y24382, Z35076, Z35077) closest to it is also common among the Komi. The Udmurts, Tatars, Chuvashs and Bashkirs have its more modern line (B226). The YSTR haplotypes of this haplogroup in the Komi and Udmurts are closer to each other than to the Khanty samples. The presence among the Khanty and Komi of two haplogroups, N1a2b2a1 and N1a2b2b1~, with ethnospecific terminal SNPs and different haplotypes indicates their inheritance from fairly ancient common ancestors, most likely part of the early Ugric population of these territories.

Thirteen samples of the Kazym Khanty belong to the haplogroup N1a1a1a1a2a1c1~ (Y13850, Y13852). Seven of them have the surname Pyak, which is Nenets in origin, referring to the Forest Nenets. All seven of these samples have very close haplotypes and are descendants of a relatively recent common Nenets ancestor. In the questionnaires of these men, who consider themselves Khanty, Nenets ancestors were indicated on the paternal line with different depths. The remaining six men of this haplogroup differ in haplotypes from the Pyak genus

In our study of the Taz Nenets (Kharkov et al., 2021), it was found that all men representing the Khanty origin of the Salinder, Lar and Tibichi clans completely belong to this haplogroup. Representatives of these genera formed in the XVIII–XIX centuries in the lower reaches of the Ob as a result of the development of the Nenets large-herd reindeer husbandry and the involvement of part of the northern Khanty in it (Kvashnin, 2003). All haplotypes of the Kazym Khanty of this haplogroup differ significantly from the haplotypes of the Taz Nenets.

The other five samples of the Kazym Khanty belong to the haplogroup N1a2b1b1b1~ (B172, Z35108). All previously surveyed Nenets men from the Vanuito phratry belonging to the Vanuito, Puiko and Yaungat clans, and the Purungui clan of Khanty origin, belong to it. Four samples of the Khanty differ in haplotypes from the Nenets, but one almost completely coincides with them. Such a division into haplotypes specific to the Khanty and close to the Nenets coincides with the data on the haplogroup N1a1a1a1a2a1c1~. It is obvious that the gene pool of the Kazym Khanty includes precisely the variants of these haplogroups of Khanty origin, but relatively recently marriages were also made with the Forest Nenets. The absence of these haplogroups in the Russkinskaya Khanty is in good agreement with the data on the distribution of IBD blocks and the coefficient of genomic inbreeding

The distribution of various haplogroups of the N clade of the Y-chromosome in the studied populations is in good agreement with the frequency of the Ugric genetic component. Phylogenetic analysis of Y-chromosomal sublines and haplotypes of various haplogroups of the N clade shows that the center of origin and distribution of the carriers of the Ugric component in Southern, Western Siberia and Eastern Europe is the territory of modern Altai and Sayan Mountains. The obtained results are well comparable with the data of ethnology, anthropology and linguistics on the contribution of the Uralic component to the formation of various peoples of the Altai-Sayan and the historical areas of Ugric and other languages of the Uralic language family.

**Fig. 3.**
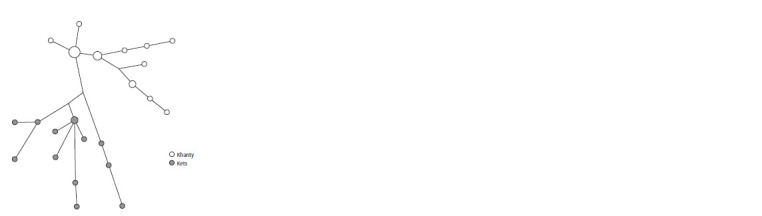
Median network of YSTR haplotypes of haplogroup Q1b1a3b1a2~ in Khanty and Kets.

Almost 40 % of men from Kazym belong to the haplogroup Q1b1a3b1a2~ (Z35974 xB32, B33, Z35993). The lineage Q1b1a3b1a2~ (B33, Z35991) specific to the Kets population is closest to it. In addition to the Kets, this variant also prevails among the Selkups from the Tomsk Region and the Krasnoyarsk Region. A more distant line Q1b1a3b1a~ (B30, YP1693 xZ35991) is common in Tuvan populations, with a maximum frequency in the eastern mountainous regions of Tuva (up to 25 %). Khanty samples show a specific haplotype spectrum with a recent founder effect that is not observed in the Kets (Fig. 3).

The distribution of these sublines in the populations of the Khanty, Kets, and Tuvans is in good agreement with the shares of matches in IBD blocks between them, the tSNE plot, and the distribution of the Ugric genetic component in these populations over the autosomal part of their gene pool. The presence of this lineage among the Khanty is not due to recent borrowing from other aboriginal populations (Kets and Selkups), but to the fact that it was already part of the settling ancestral groups.

Three men from the village of Russkinskaya have a completely different haplogroup of the Q clade – Q1a2b~ (M25, L716, YP1674, YP1676). This is a very rare haplogroup not found in other Siberian populations. It is presented with the maximum frequency among the ethnic Turkmens of Karakalpakstan, Iran and Afghanistan (Grugni et al., 2012; Skhalyakho et al., 2016). In most other ethnic groups, its frequency is very low. Khanty haplotypes are quite different from other populations. Most likely, the presence of this line among them is not a consequence of recent miscegenation, but is a legacy of the Ugric groups that migrated from southern Siberia and the Urals to the north.

**Fig. 4.**
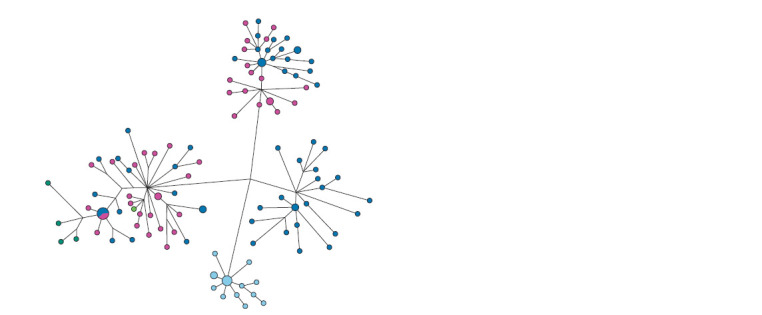
Median network of YSTR haplotypes of haplogroup R1a1a1b2-Y43850 in the Khanty, Khakas, Shors, Tuvans and Altaians. Khanty are in light blue, Khakas are in blue, Shors are in crimson, Tuvans are in dark green, Altaians are in green.

The last haplogroup, which includes 16 Khanty men from the village of Russkinskaya, is R1a1a1b2-Y43850. The haplotypes of all samples are quite close, which indicates a recent founder effect (Fig. 4).

Khanty-specific terminal SNPs are S7280, FGC687, and FGC38304. The R1a1a1b2-Y43850 variants closest to this lineage are represented with a high frequency in the Khakas and Shors, and less frequently in the Tuvans and Northern Altaians. According to YFull, this haplogroup is approximately 3800 years old. All of these patterns belong to four different lineages that split a long time ago. The age of the haplotype cluster in the Khanty was 933 years (SD = 336 years), which is approximately one and a half times less than the age of the South Siberian lines. The Khakas seok Piltir is 1469 years old (SD = 342 years) (Y39884 хY43109). The lineage of this haplogroup (Y62155.2) specific for the Biryusa Khakas seoks of Turan, Khyzyl Khaya and Shor seoks of Tartkyn, Shor-Kyzai and Kara-Shor has approximately the same age – 1315 years (SD = 227 years). The branch with a wider distribution in the Sayan-Altai populations (Y43109) is even older – 1566 years (SD = 350 years). The difference in SNP and STR among the Khakas, Shors, Tuvans, and Northern Altaians is greater than with the Khanty.

A strong heterogeneity of the studied samples of the Khanty in terms of the composition and frequencies of various haplogroups is shown. The phylogeny of various lineages of two haplogroups, N1a2b1b1 and R1a1a1b2-Y43850, indicates their South Siberian origin in the Khanty gene pool. The territory of the Sayan and Altai was the primary focus of the generation of diversity and the expansion of the number of ancestral groups of carriers of these haplogroups in Siberia. It is most likely that the distribution of most Y-chromosome haplogroups among the Khanty occurred during the initial settlement of the Ugric tribes to the north and west.

It is necessary to take into account the fact that the range of modern Khanty is located to the north of the territory of their ancestors. The West Siberian and Volga-Ural regions were the place of secondary generation of diversity, but not the formation of the N1a2 haplogroup itself. At the moment, there is no final opinion regarding the place of formation of the ethnoi of the Uralic language family, but numerous data, including the results of studies of the phylogeny and phylogeography of clade N haplogroups, point to Southern Siberia. Linguistic paleontology points to the Proto-Ural ecological area as a territory limited in the west by the Ural Range, in the north by approximately the Arctic Circle, in the east by the area of the lower reaches of the Angara and Podkamennaya Tunguska and the middle reaches of the Yenisey, in the south by approximately the modern southern border of the West Siberian taiga from the northern foothills of the Sayan and Altai to the lower reaches of the Tobol and the Middle Urals inclusively (Napolskikh, 2018).

## Conclusion

Thus, the gene pool of the two Khanty populations is a heterogeneous set of Y-chromosome haplogroups, but very similar in autosomal markers. The expanded composition of terminal SNPs for the identified haplogroups made it possible to describe in detail and clarify the differences in the phylogeny and structure of individual ethnospecific sublines, to determine their relationship, and traces of population expansion in the Khanty gene pool. The results of a comparative analysis of male samples indicate a close genetic relationship of the Khanty with the Altai-Sayan Khakas and Tuvans, as well as with the Nenets, Komi, Udmurts and Kets. The specificity of haplotypes and the detection of various terminal SNPs indicate that the Khanty did not come into contact with other ethnic groups for a long time. The only exception is the Nenets, which included many Khanty clans. For the northern population of the Kazym Khanty, Y-chromosomal lines show a small contribution of the Forest Nenets.

The results obtained do not contradict the generally accepted versions of the Khanty ethnogenesis, but allow us to take a fresh look at this process. The main factor in the formation of the Khanty gene pool was their territorial genetic isolation and later mixing with the newcomer Samoyed population, which, when switching to tundra reindeer husbandry, led to a strong demographic growth of their clans as part of the Nenets. The relatively low genetic diversity in autosomal SNPs and the rather high level of inbreeding in the Khanty confirm this. New information about the structure of the Khanty gene pool is an important addition to the existing anthropological, archaeological, ethnological and linguistic data on their formation and kinship with other peoples.

## Conflict of interest

The authors declare no conflict of interest.
